# Increased Productivity of a Cover Crop Mixture Is Not Associated with Enhanced Agroecosystem Services

**DOI:** 10.1371/journal.pone.0097351

**Published:** 2014-05-21

**Authors:** Richard G. Smith, Lesley W. Atwood, Nicholas D. Warren

**Affiliations:** Department of Natural Resources and the Environment, University of New Hampshire, Durham, New Hampshire, United States of America; Agroecological Institute, China

## Abstract

Cover crops provide a variety of important agroecological services within cropping systems. Typically these crops are grown as monocultures or simple graminoid-legume bicultures; however, ecological theory and empirical evidence suggest that agroecosystem services could be enhanced by growing cover crops in species-rich mixtures. We examined cover crop productivity, weed suppression, stability, and carryover effects to a subsequent cash crop in an experiment involving a five-species annual cover crop mixture and the component species grown as monocultures in SE New Hampshire, USA in 2011 and 2012. The mean land equivalent ratio (LER) for the mixture exceeded 1.0 in both years, indicating that the mixture over-yielded relative to the monocultures. Despite the apparent over-yielding in the mixture, we observed no enhancement in weed suppression, biomass stability, or productivity of a subsequent oat (*Avena sativa* L.) cash crop when compared to the best monoculture component crop. These data are some of the first to include application of the LER to an analysis of a cover crop mixture and contribute to the growing literature on the agroecological effects of cover crop diversity in cropping systems.

## Introduction

Cover crops are typically sown within annual crop rotations to protect soil from erosion or provide other agroecosystem services such as building soil fertility and organic matter, retaining nutrients, or suppressing weeds during periods when cash crops are not actively growing [1]–[3]. Typically, these crops are sown as monocultures or simple graminoid-legume bicultures [2]; however, there is increasing interest among growers and researchers in investigating whether there may be additional benefits to growing cover crops in more species-diverse mixtures [4]. While there has been a large number of studies examining the role that crop diversity (including the use of cover crops within diversified rotations) plays with respect to specific agroecosystem services [5]–[7], few studies have examined the role of cover crop diversity explicitly (but see [4], [8]).

There are a number of reasons to expect that a more diverse cover crop mixture might confer enhanced agroecosystem services relative to a monoculture or simple biculture. First, a wide range of plant species can be used as cover crops, including species from the graminoid, legume, brassica, and other broad-leaved families [9]. While each individual species may excel at one or a few services, no species is capable of providing all of the possible services and at the magnitudes likely necessary for substantive benefits to the agroecosystem. Thus, a cover crop mixture that contains a diversity of species, each differing in functional traits (e.g., biological N-fixation, root system, growth rate, tissue C:N, floral display, LAI, etc.) could be expected to provide a greater diversity of services relative to a monoculture or a two-species cover crop community.

Second, there is often a positive relationship observed between cover crop productivity and its effectiveness for weed suppression [10], [11]. Diversity-productivity theory suggests that increased productivity associated with species diversity is due to more efficient resource use [5], [12]. Thus, diverse cover crop communities should be expected to produce more biomass than cover crop monocultures. Diverse cover crop communities should also be expected to be more weed suppressive because fewer resources are left available to support weed establishment and growth [13], and compared to monocultures, they may result in a broader spectrum of allelopathic activity toward various weed species or other soil environment modifications that enhance weed suppression [13], [14].

Cover crop monocultures are subject to the same risks associated with variable growing conditions as are cash crop monocultures [15]. Therefore, diverse cover crop communities that contain multiple species with differing soil and environmental optima should be expected to be less variable in terms of overall stand productivity and function over space and time than cover crop monocultures or simple bicultures [4], [16].

There are also reasons why a diverse cover crop mixture may be less desirable to a farmer than a mono- or biculture. These include increased costs for cover crop seed [17]; difficulty in establishing and managing complex mixtures, particularly if species have very different seed sizes, growth rates, life histories, or termination requirements [8], [14]; and the possibility of antagonistic interactions between particular cover crop species or other components of the cash crop rotation [2]. Given the theoretical and practical arguments both for and against diverse cover crop mixtures, there is a clear need for additional research that addresses how diversified cover crop mixtures affect the myriad of agroecosystem functions and services that underpin the sustainability of agriculture.

Recently, Wortman et al. [4] reported land equivalent ratio (LER) and stability indices for multi-species mixtures of legume and brassica cover crops. Their study provided the first evidence available that cover crop mixtures are capable of over-yielding (i.e., LER>1) relative to the component species grown as monocultures. While these data help to confirm some of the suspected benefits of multi-species cover crop plantings, the study was limited to only two plant functional groups, legumes and brassicas. They also did not report on other agroecosystem services beyond productivity and stability, such as weed suppression. Also unknown are the effects diverse cover crop mixtures have on the growth of subsequent cash crops which would be planted after the cover crop mixture is terminated. Thus, the generality of the findings reported by Wortman et al. [4], and the potential for diverse cover crop mixtures to provide agroecosystem services relative to weed suppression and cash crop productivity remain unclear.

The objective of this study was to determine whether a mixture containing a functionally diverse group of spring-sown cover crops representing four plant families (Polygonaceae, Brassicaceae, Fabaceae, and Poaceae) could provide enhanced agroecosystem services relative to the component cover crops grown in monoculture. Specifically, we were interested in testing the following hypotheses:

1. A diverse cover crop mixture will be more productive than the most productive component crop grown in monoculture.
2. A diverse cover crop mixture will suppress weeds better than the most suppressive component crop grown in monoculture.
3. A diverse cover crop mixture will be more stable, in terms of biomass production and weed suppression, than the component crops grown in monoculture.
4. The biomass production of a subsequent crop will be higher following a cover crop mixture compared to monocultures of the component crops.

## Materials and Methods

### Site Description

The experiment was conducted at the University of New Hampshire Kingman Research Farm in Madbury, NH (43^o^11′N 70^o^56′W). Dominant soil type at this site is a Hollis-Charlton fine sandy loam (Hollis = loamy, mixed, mesic Entic Lithic Haplorthods; Charlton = coarse-loamy, mixed, mesic Entic Haplorthods) [18]. Mean monthly precipitation during the growing season (May–September) ranges from 89.9 to 107.4 mm and high and low temperatures range from 21 to 28°C and 7 to15°C, respectively. For several years prior to the experiment the site had been under a conventionally managed vegetable-winter rye (*Secale cereal* L.) cover crop rotation as part of a squash and pumpkin (Cucurbitaceae) breeding program.

### Experimental Design

The experiment was conducted in 2011 and again in 2012 at an adjacent site. The experimental design both years was a randomized complete block with eight cover crop treatments, each replicated four times. The cover crop treatments were monocultures of buckwheat (*Fagopyrum esculentum*), mustard (*Brassica juncea*), sorghum-sudangrass (*Sorghum bicolor* var. sudanense), cereal rye (*Secale cereale*), hairy vetch (*Vicia villosa*) (2011 only) or field pea (*Pisum sativum*) (2012 only), and a mixture of all five species in which individual seeding rates were 20% of the monoculture rate (Table 1). A mixture of all five species in which individual seeding rates were 100% of the monoculture rate and a weedy fallow treatment in which no cover crops were planted were also included in the experimental design; however, these treatments were not germane to the present study and were therefore excluded from this analysis. Cover crop species were chosen to represent a diversity of plant families (i.e., Polygonaceae, Brassicaceae, Poaceae, and Fabaceae) corresponding to different plant functional groups (broadleaf forbs, C4 and C3 grasses; nitrogen-fixers; [19]). Field pea was substituted for hairy vetch in 2012 due to poor hairy vetch performance in 2011. In 2011, the experimental units were 2.5 m by 4 m, and in 2012 they were 4 m by 4.9 m. Prior to establishing each run of the experiment, the site was moldboard plowed and the seedbed was prepared using a Perfecta II field cultivator (Unverferth Equipment, Kalida, OH). Cover crops were broadcast seeded by hand in late spring (14 June 2011 and 19 June 2012) and seeds were incorporated into the soil with a rake.

**Table 1:** Seeding rates of the cover crops used to create the mixture and component monoculture treatments in 2011 and 2012.

Cover crop treatment	Species	Family	Seeding rate
			(kg ha^−1^)
Buckwheat	*Fagopyrum esculentum*	Polygonaceae	67.2
Hairy vetch (2011 only)	*Vicia villosa*	Fabaceae	44.8
Field pea (2012 only)	*Pisum sativum* ‘maxum’	Fabaceae	224
Mustard	*Brassica juncia* ‘Pacific gold’	Brassicaceae	6.72
Sorghum-sudangrass	*Sorghum bicolor* x *S. Bicolor* var. sudanese	Poaceae	33.6
Cereal rye	*Secale cereale*	Poaceae	112
Mixture	All	All	All at 20% of full-rate

Monoculture treatments were seeded at recommended rates for each species (Table 1). To evaluate cover crop mixture effects relative to the component species, we used a substitutive approach (i.e., proportional replacement design) such that seeding rates for each species in the mixture were proportional to their monoculture rate [20]. Therefore, the seeding rates for individual species in the mixture were determined by dividing each recommended seeding rate by the total number of species in the mixture (i.e., five). This approach minimizes potentially confounding effects of a higher overall seeding rate in the mixture and preserves the ability to use well established intercropping indices such as the LER [4], [20]. No fertilizers or pesticides were applied to the experimental plots during the duration of the experiment.

### Cover Crop Productivity

We quantified cover crop productivity for all treatments using two metrics: total aboveground biomass per unit area (dry weight, kg ha^−1^) and the LER, which is traditionally used to evaluate the productivity of crop mixtures relative to component monocultures [4], [21]. Both metrics were assessed by harvesting cover crop biomass at the soil surface from six 0.5 by 0.5 m quadrats located semi-randomly within each treatment replicate (the replicate was divided into six zones and one quadrat was positioned randomly in each zone). The six quadrat locations from which cover crop biomass was measured corresponded to the location of four “surrogate weed” subplots (described below) and two additional locations, all of which were located at least 0.5 m from the edge of the plot. The cover crop biomass harvest occurred at 43 (2011) and 72 (2012) days after the cover crop treatments were planted. The harvest in the 2012 study was delayed relative to the 2011 study in an effort to generate higher overall cover crop biomass. Harvested biomass was separated to species, dried at 65°C to constant biomass, and weighed to the nearest 0.01 g. Plot-level cover crop biomass (shoot dry weight) was then calculated for each treatment replicate by averaging the six subsamples. It is important to note that difference in harvest time (and substitution of legume species) between years does not impact our ability to quantify effects of the mixture relative to its component species, but does restrict our ability to make statements regarding the importance of climate factors as drivers of between-year differences in cover crop performance.

Plot-level cover crop biomass data were used to calculate the LER for the mixture, which represents the amount of land area that would be required to grow the individual component species as monocultures so as to achieve the same level of productivity as was attained in the mixture [21]. The LER is calculated as:
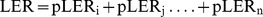
where pLER*_i_* is the partial LER of cover crop species *i*, pLER*_j_* is the partial LER of cover crop species *j*, and so forth for all *n* number of cover crop species present in both the mixture and monoculture. The partial LER of a given cover crop species (*i*…*n*) is calculated as:



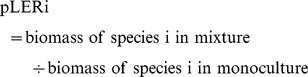



A total LER for a mixture>1 indicates that more land area would be required to grow the cover crops as monocultures than growing an equivalent biomass using a mixture (i.e., the mixture “over-yielded” relative to the component monocultures). Conversely, a LER <1 indicates that less land would be necessary for monocultures than for a mixture to achieve an equivalent biomass yield (i.e., the mixture “under-yielded” relat’ive to the component monocultures). The partial LER for individual species can be used to compare their relative contribution to the total LER. In our case, because the mixture contained five species, the pLER for each species would be 0.20 in the absence of any interspecific interactions. Thus, the pLER indicates whether each species was positively (i.e., facilitation, when pLER>0.20) or negatively (i.e., antagonism, when pLER <0.20) affected by the other mixture components relative to its performance in monoculture [4]. Partial and total LERs were calculated at the block-level to enable statistical analysis (see below).

### Weed Suppression

We used two approaches to quantify how the cover crop mixture and component monocultures affected the agroecosystem function of weed suppression. First, weeds that emerged from the soil seed bank (i.e., “ambient weeds”) were harvested at the same time and from the same six quadrats used to collect cover crop biomass samples. Weeds were also sorted to species, dried at 65°C to constant biomass, and weighed to the nearest 0.01 g. Second, we also established a “surrogate weed community” within four of the six 0.5 m by 0.5 m subplots located within each experimental unit. The surrogate weed community consisted of a total of 50 seeds made up of five crop plant species (Table 2). The purpose of the surrogate weeds was to create a uniform weed density and composition to more thoroughly assess competitive effects of the cover crop treatments. The rationale for planting crop species, as opposed to “weed” species, was to differentiate between seeds we added from those that emerged from the ambient seed bank and to ensure rapid germination and growth. With the exception of *Helianthus annuus*, which is in the Asteraceae family, the surrogate weed species were chosen to represent the same plant families included in the cover crop treatments. Seeds of the surrogate weed community were sown by hand into each subplot at two times (“early” to simulate weeds emerging at the same time as the cover crop, and “late” to simulate weeds emerging several weeks later). Within each plot, two subplots were designated as “early” and two were designated as “late”. Surrogate weed communities were planted on 17 June 2011 and 20 June 2012 for “early” subplots and 30 June 2011, and 12 July 2012 for “late” subplots. Subplot locations were marked with stakes to facilitate relocation at the time of sampling. Surrogate weed biomass was collected at the same time and in the same manner as the cover crop and ambient weed biomass.

**Table 2:** Crop species used to create “surrogate weed community” subplots in 2011 and 2012.

Surrogate weed	Species	Family	Density
			(No. m^−2^)
Sorrel	*Rumex sanguineus*	Polygonaceae	40
Field pea (2011)	*Pisum sativum*	Fabaceae	40
Red clover (2012 only)	*Trifolium pratense* ‘mammoth’	Fabaceae	40
Canola	*Brassica napus*	Brassicaceae	60
Wheat (2011 only)	*Triticum aestivum*	Poaceae	40
Oats (2012 only)	*Avena sativa*	Poaceae	40
Sunflower	*Helianthus annuus* ‘Zebulon’	Asteraceae	20

Analysis of cover crop mixture and component monoculture effects on the abundance of “ambient” weeds were based on samples collected from the two quadrats that did not contain surrogate weeds and the two quadrats that contained the “late” surrogate weed subplots. The “late” subplots were included in the analysis of the ambient weeds because emergence of surrogate weeds from those quadrats was effectively zero (data not shown). Cover crop treatment effects on surrogate weed abundance were thus restricted to the two “early” subplots within each replicate.

### Stability

Cover crops that exhibit variable performance (i.e., are not stable) across space or time are not likely to be adopted by farmers, who often tend to be risk averse. We assessed spatial (plot to plot) and temporal (year to year) stability of the mixture and component treatments using the approach described by Tilman [22] and Wortman et al. [4]. Stability was assessed for both cover crop biomass production and ambient weed suppression by calculating the coefficient of variation (CV) for each cover crop treatment pooled across replications (n = 4) and years (n = 2). The CV was calculated as the standard deviation of cover crop biomass (or weed biomass) divided by the average cover crop biomass (or weed biomass). A lower CV indicates lower variability and hence greater stability in biomass production or weed suppression [22].

### Carryover Effects on the Productivity of a Subsequent Crop

We examined carryover effects of the 2012 treatments by quantifying growth of a subsequent oat (*Avena sativa*) crop. The 2012 study site was cut with a sickle bar mower on 16 November 2012 to a height of 6 cm, and residues were allowed to remain within the plot over winter. In spring (20 May 2013), the plots were georeferenced and the entire site was chisel plowed. Following tillage, the field was prepared using a Perfecta II field cultivator (Unverferth Equipment, Kalida, OH), and oats were broadcast at a rate of 168 kg ha^−1^ on 31 May 2013. No fertilizer or herbicides were applied. On 30 July 2013 the location of the boundaries corresponding to the previous year’s cover crop treatment plots were geolocated and oat biomass was harvested from two 0.5 by 0.5 m quadrats placed within 1.25 m of the center of each plot. Weed species were removed from the harvested oats and the biomass was dried at 65°C to constant biomass, and weighed to the nearest 0.01 g. The oat response was not initially an objective of the study, and thus was not implemented following the 2011 treatments.

### Statistical Analyses

Cover crop, ambient weed, and surrogate weed dry biomass data were analyzed with the MIXED procedure in SAS (SAS Institute, Cary, NC, USA). The factors cover crop treatment, year, and the treatment x year interaction were all considered fixed effects. The block effect was considered random. The oat biomass data were analyzed as above, but without including the year and treatment x year interaction factors in the model. If significant treatment differences were detected, pairwise comparisons were made using least squares means. Distributions of the raw data did not deviate significantly from normal but were heteroscedastic. Transformations did not result in homogeneity of variance and tended to result in departures from a normal distribution; therefore, data were analyzed untransformed and presented as box-plots to enable visualization of the distribution of responses. One sample *t*-tests were used to determine whether the total LER of the mixture and partial LERs of the mixture components differed from 1 and 0.20, respectively. The weedy fallow treatment and mixture treatment in which all species were seeded at 100% of the full rate were not included in any of the analyses.

## Results

### Productivity

**Figure 1:**
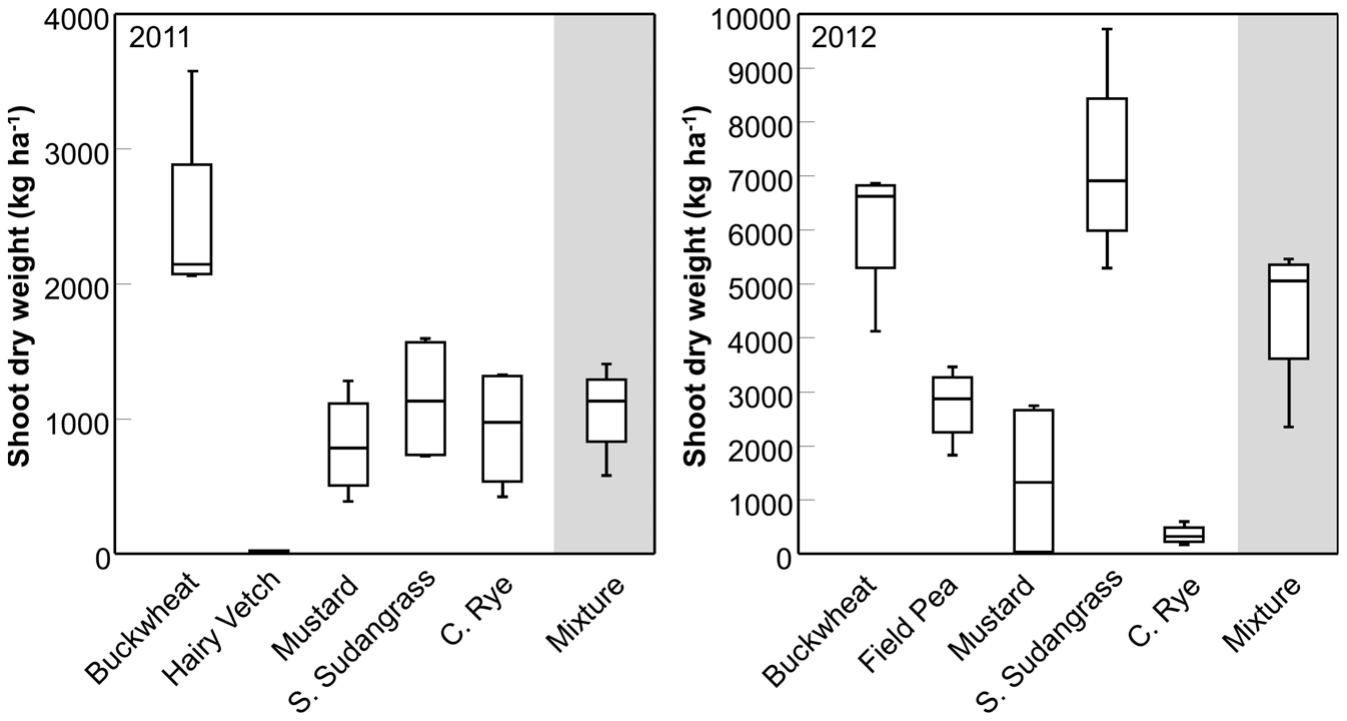
Productivity of a cover crop mixture and component monocultures. Box plots showing variation around the median for shoot dry weight of five cover crops grown in monoculture and a mixture containing all five species in 2011 and 2012. The line within the box represents the median; the box represents 50% of the data; whiskers represent the 10^th^ and 90^th^ percentiles; n = 4.

Analysis of the cover crop biomass dry weight data indicated a significant treatment by year interaction (treatment x year: F_5,33_ = 11.76, *P*<0.0001); therefore, the data from each year were analyzed separately. The subsequent analyses indicated that cover crop treatment effects were significant in both years (2011: F_5,15_ = 12.65, *P*<0.0001; 2012: F_5,15_ = 16.28, *P*<0.0001). In 2011, the treatment effects were driven primarily by buckwheat (2,478±363 kg ha^−1^, mean ±1 SE) and hairy vetch (15±4 kg ha^−1^), which produced significantly higher and lower biomass than the other five treatments, respectively (*P*<0.05). The biomass of the mixture (1,062±174 kg ha^−1^) did not differ from the mustard, sorghum-sudangrass, or cereal rye monocultures (Figure 1). In 2012, there was more differentiation in biomass between the treatments. Biomass of the sorghum-sudangrass monoculture (7,200±926 kg ha^−1^) was significantly higher than all the other treatments (*P*<0.05), except the buckwheat monoculture. Biomass of the mixture (4,476±720 kg ha^−1^) was not significantly different from the buckwheat or field pea monocultures, but was higher than the mustard and cereal rye monocultures (Figure 1).

**Figure 2:**
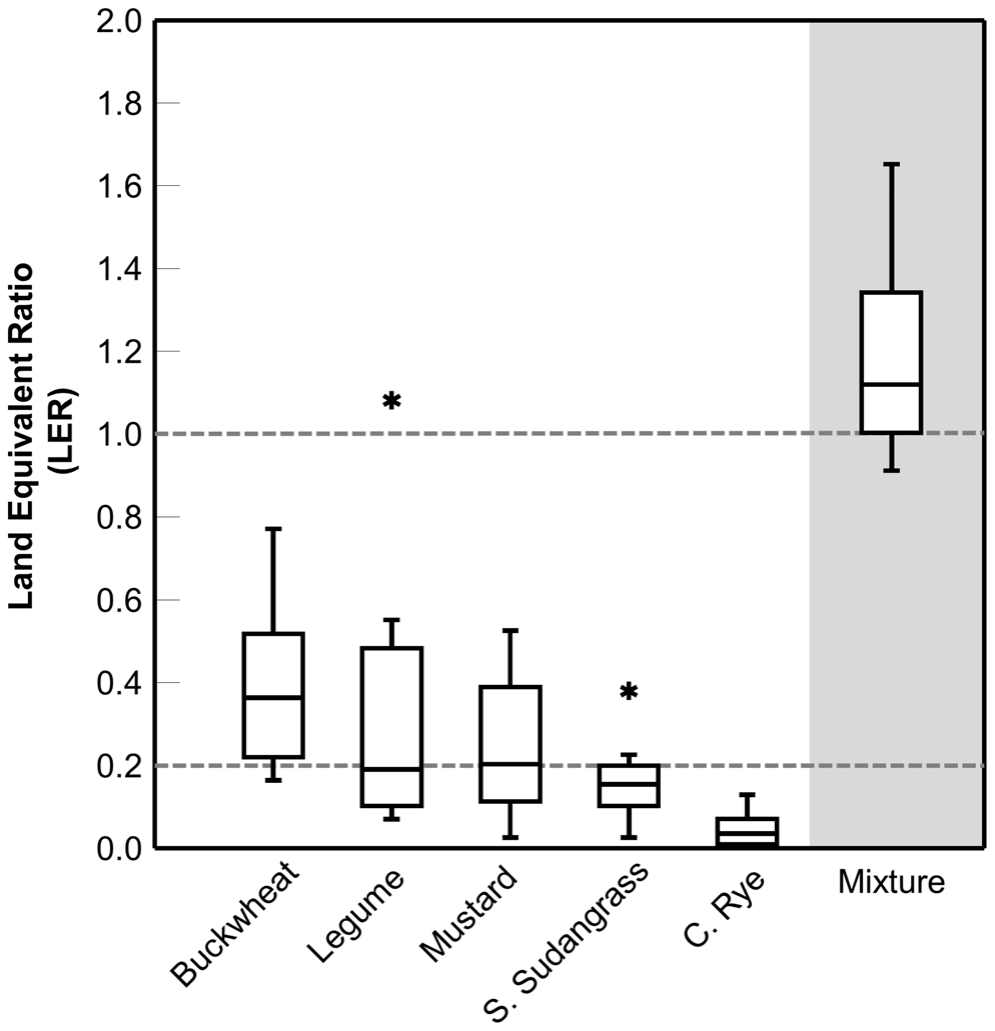
Land equivalent ratio (LER) of the mixture. Box plot showing variation around the median for the partial (individual species contributions) and total LER of the cover crop mixture across the two study years. The grey dotted lines at 0.2 and 1.0 indicate “break even” points above which partial and total LER indicate over-yielding, respectively. The line within the box represents the median; the box represents 50% of the data; whiskers represent the 10^th^ and 90^th^ percentiles; asterisks indicate outliers; n = 8.

In 2011 the mean LER of the mixture was 1.26, while in 2012 it was 1.12. Pooled across both years, the LER for the mixture was significantly greater than 1 (LER = 1.19±0.09; *t*-test, *P* = 0.035), indicating the mixture over-yielded relative to the component monocultures (Figure 2). This result means the mixture resulted in more efficient use of the land than the alternative of growing the component species as monocultures [4]. Investigation of the partial LERs indicated that only buckwheat had a pLER greater than 0.2, suggesting this species contributed most to the over-yielding response (pLER = 0.39±0.07; *t*-test, *P* = 0.017), and that its growth may have been facilitated by interspecific interactions. Conversely, only one species, cereal rye, had a pLER that was less than 0.2 (pLER = 0.05±0.02; *t*-test, *P*<0.001), suggesting that its growth may have been limited by the other species in the mixture (Figure 2). The pLER for the other three species did not differ from 0.2 (*P*>0.05).

### Weed Suppression

**Figure 3:**
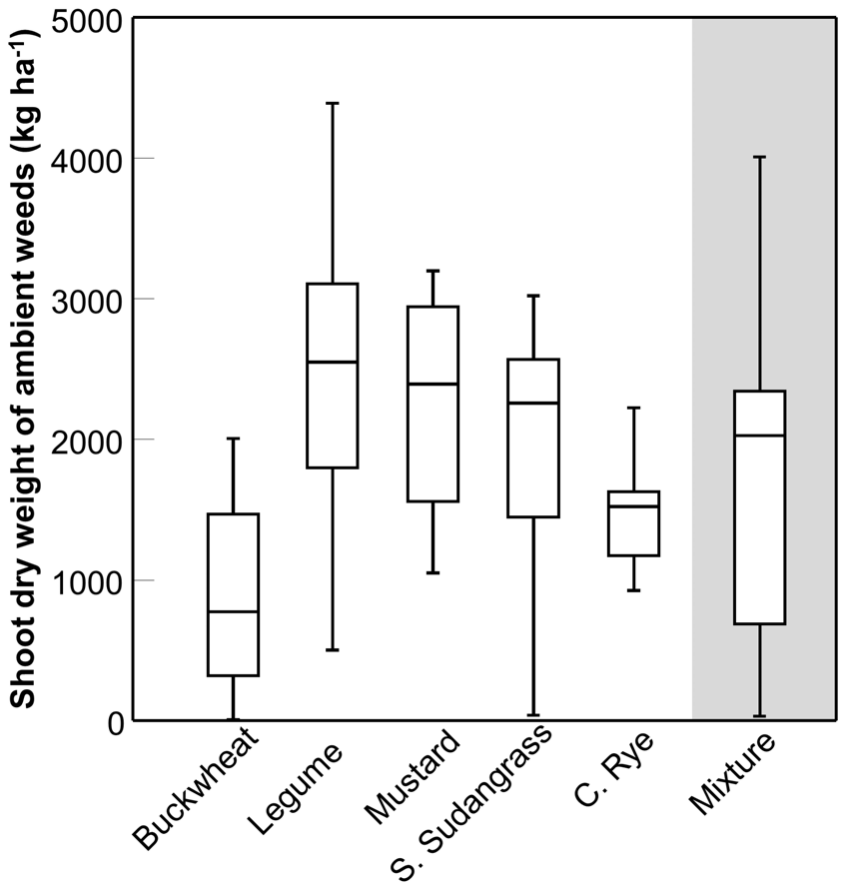
Biomass of weeds that emerged from the ambient weed seed bank. Box plots showing variation around the median for ambient weed biomass in five cover crops grown in monoculture and a mixture containing all five species across the two study years. The line within the box represents the median; the box represents 50% of the data; whiskers represent the 10^th^ and 90^th^ percentiles; n = 8.

The ambient weed biomass present at harvest differed by year (F_1,13_ = 21.27, *P*<0.0001) and cover crop treatment (F_5,33_ = 4.75, *P* = 0.0022), but there was no interaction between year and treatment. Across the two years, ambient weed biomass was lower in buckwheat monoculture plots compared to legume, mustard, and sorghum-sudangrass monocultures (*P*<0.05). Weed abundance in the mixture was not significantly different from buckwheat or other component monocultures (Figure 3).

**Figure 4:**
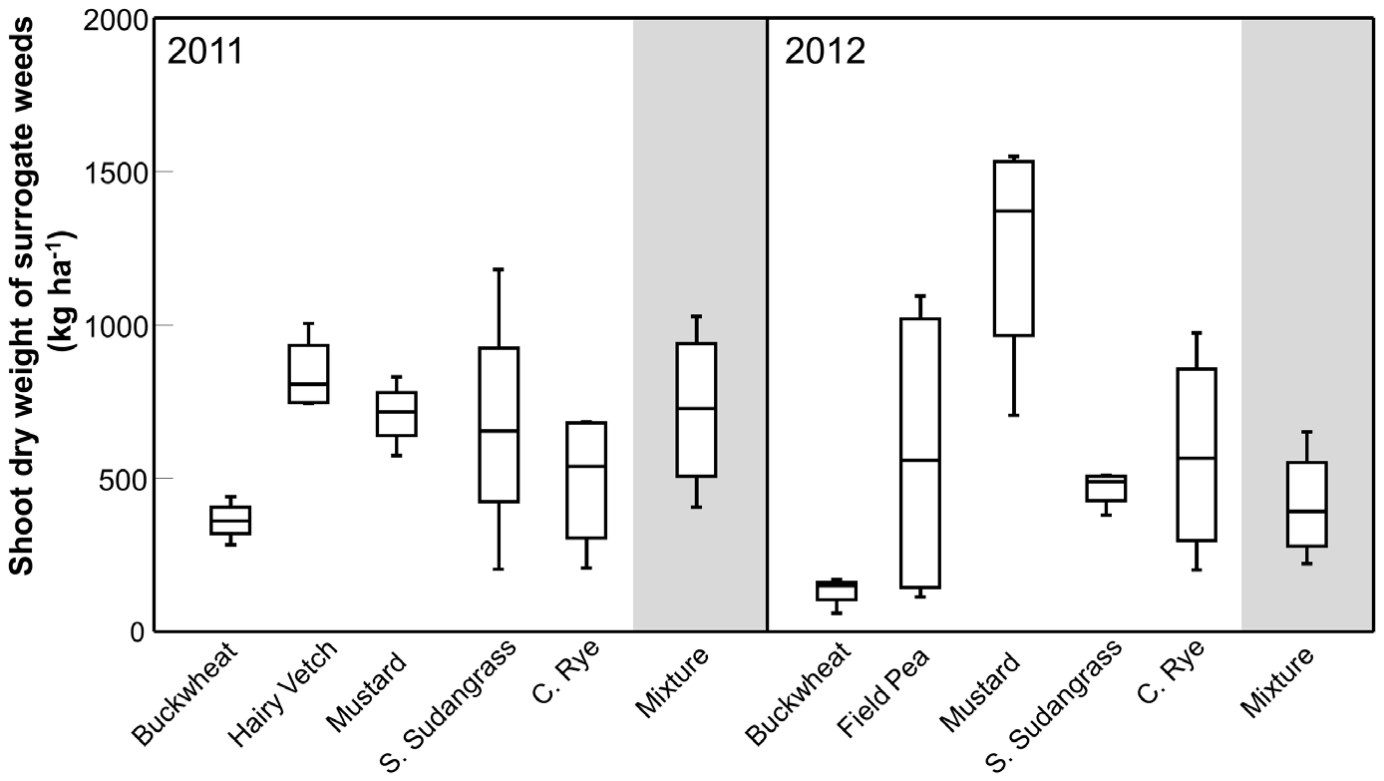
Biomass of surrogate weeds. Box plots showing variation around the median for surrogate weed biomass in five cover crops grown in monoculture and a mixture containing all five species in 2011 and 2012. The line within the box represents the median; the box represents 50% of the data; whiskers represent the 10^th^ and 90^th^ percentiles; n = 4.

Surrogate weed response indicated an interaction between year and cover crop treatment (F_5,33_ = 3.22, *P* = 0.0178); therefore, the data were analyzed separately for each year. In 2011, surrogate weed biomass tended to be lower in the buckwheat monoculture, but differences among treatments were not statistically significant (F_5,15_ = 2.48, *P* = 0.079). In 2012, treatment differences were significant (F_5,15_ = 6.05, *P* = 0.003) and were driven primarily by the mustard monoculture. Surrogate weed biomass was higher in the mustard monoculture than any of the other monocultures or the mixture (*P*<0.05). Surrogate weed biomass in the mixture was not significantly different from the other four monocultures, despite a trend toward lower biomass in the buckwheat monoculture (Figure 4).

### Stability of Productivity and Weed Suppression

**Figure 5:**
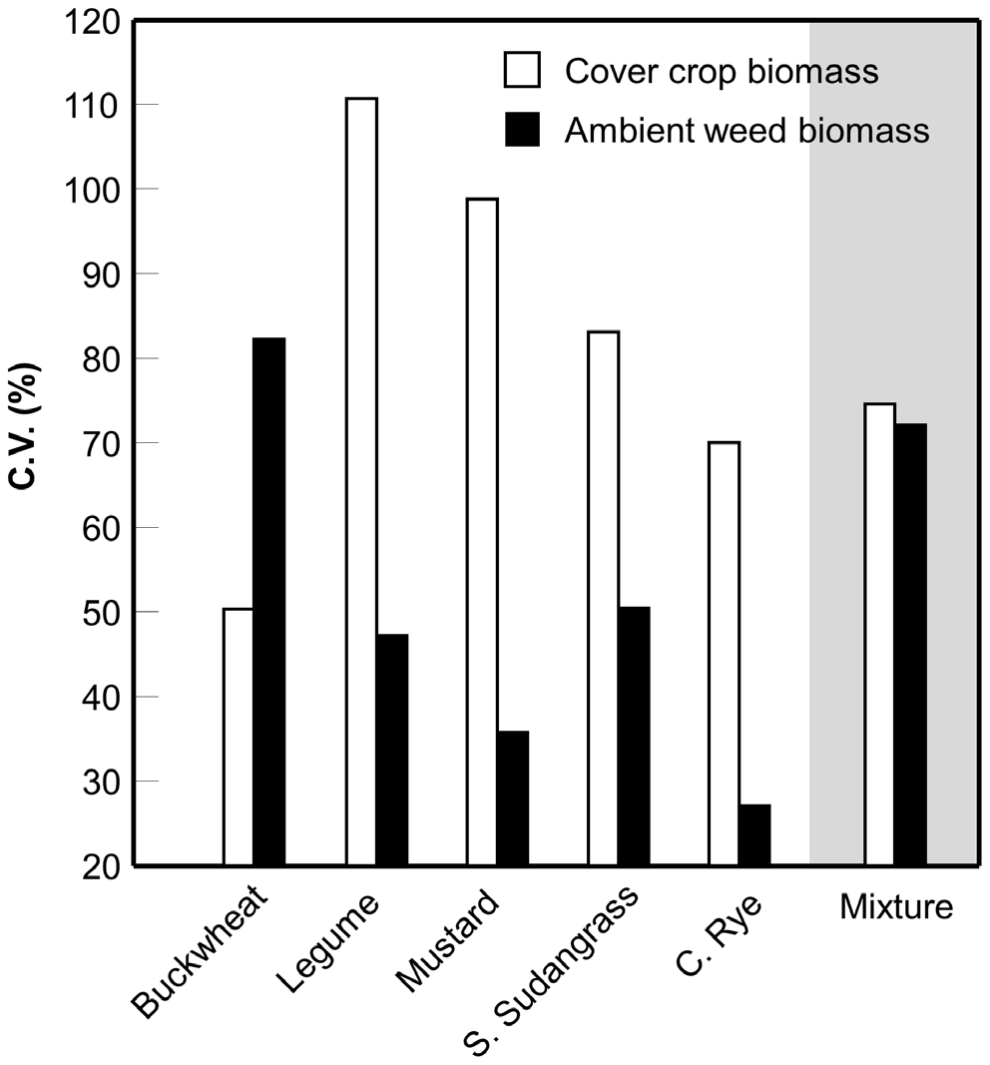
Variability in weed suppression in space and time. Coefficient of variation (CV) calculated across replicates (n = 4) and years (n = 2) for cover crop and ambient weed biomass in each cover crop monoculture and a mixture containing all five cover crop species.

The CV was used as a measure of the relative stability of the different cover crop treatments in terms of their productivity and weed suppression. Buckwheat (CV = 50%) and cereal rye (CV = 70%) monocultures had the least variable biomass production across replicates and years. The mixture (CV = 75%) was also less variable in space and time than legume, mustard, and sorghum-sudangrass monocultures (Figure 5). With respect to weed suppression, the cereal rye monoculture had the least variable weed abundance (CV = 27%). In contrast, weed abundance was most variable in the buckwheat monoculture and mixture treatments (buckwheat CV = 82%; mixture CV = 72%; Figure 5).

### Carryover Effects on Subsequent Crop Productivity

**Figure 6:**
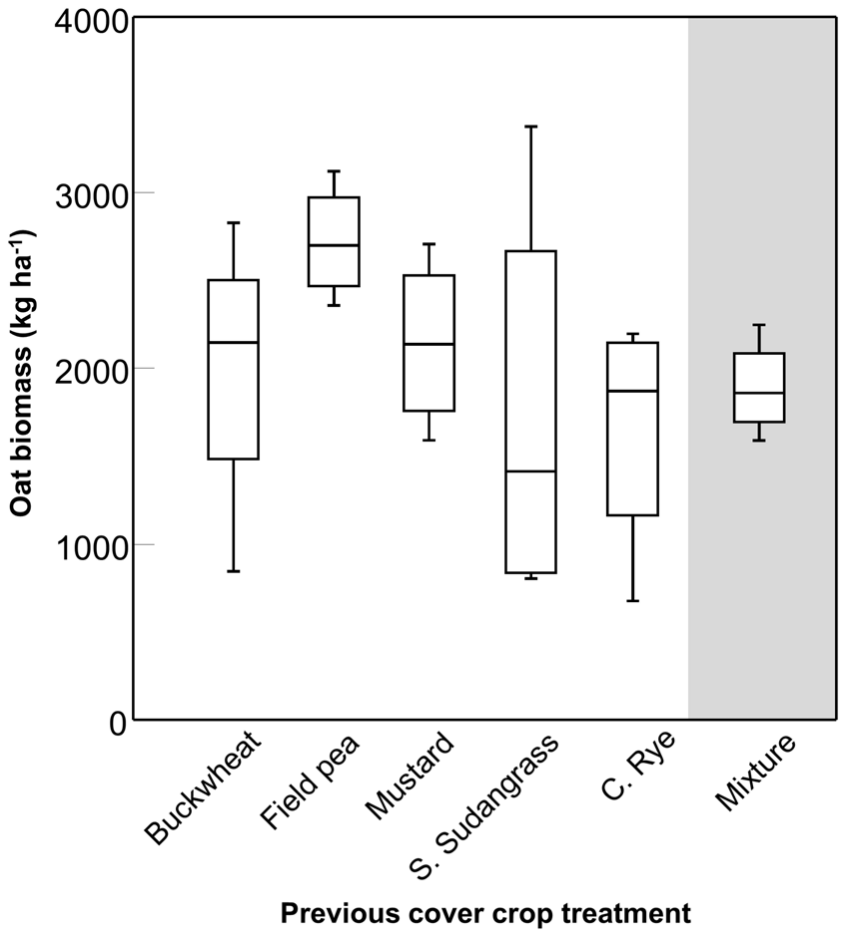
Carryover effects on oat growth. Box plots showing variation around the median for biomass of an oat phytometer sown in 2013 on plots that were previously sown with five cover crops grown in monoculture and a mixture containing all five species. The line within the box represents the median; the box represents 50% of the data; whiskers represent the 10^th^ and 90^th^ percentiles; n = 4.

An oat crop was planted uniformly across the 2012 study site to quantify the potential carryover effects of the previous cover crop treatments on oat productivity. Oats following field pea monoculture tended to have higher biomass than following the other treatments, including the mixture (Figure 6), but the effect was not statistically significant (*P* = 0.168).

## Discussion

The objective of this study was to assess whether a cover crop mixture containing five species from four different plant families would provide enhanced agroecosystem services relative to the same species grown as monocultures. The services we measured included cover crop productivity, weed suppression, biomass stability, and carryover effects on the productivity of a subsequent crop. Although our study included five different cover crop species, our intent was not to examine all possible levels of diversity. Instead, we were interested in the extreme ends of the diversity gradient (i.e., monocultures vs. a mixture of all species), assuming these extremes would correspond to the greatest differences in agroecosystem functional response [23], [24]. The time period for our study was designed to simulate a summer fallow period that might precede a late summer/fall or subsequent spring cash crop. Thus, our results may not apply to all possible cover crop niches (e.g., fall-sown cover crops), cover crop species, or cover crop combinations.

While our data do not support the hypothesis that a diverse cover crop mixture would produce more biomass on a per unit area basis than the most productive component crop grown in monoculture, we did observe an increased biomass yield (i.e., over-yielding) with the mixture relative to the component monocultures. This seemingly contradictory result deserves further clarification. With regard to productivity responses in plant biodiversity studies, Schmid et al. [25] distinguish between ‘over-yielding’ and ‘transgressive over-yielding’. Over-yielding occurs when the biomass production of the mixture is greater than the average monoculture yield [25]. Similar to Wortman et al. [4], we observed this type of response in our mixture treatments, as indicated by LER values greater than 1.0 in both 2011 and 2012 (Figure 2). In contrast, transgressive over-yielding occurs when biomass of the mixture is greater than that produced by the most productive monoculture [25]. Like Wortman et al. [4], we did not observe biomass production in the mixture to be higher than the most productive monoculture treatment (i.e., buckwheat in 2011 and sorghum-sudangrass in 2012; Figure 1). This response suggests higher biomass yields may not be a realistic outcome of cover crop mixtures constructed using a substitutive approach.

The over-yielding observed in our study was attributed mainly to buckwheat, which produced proportionately more biomass per unit area when grown in mixture than in monoculture. This was confirmed by a partial LER that exceeded 0.20. The only species that appeared to be negatively affected by being grown in mixture was cereal rye. Since that crop is typically sown in the fall, it was not unexpected for it to be less competitive when sown in the spring [26]. To our knowledge, ours is the first study to assess yield response of cover crop mixtures containing buckwheat, sorghum-sudangrass, and cereal rye using the LER. Therefore, we are not certain that the partial LER values reported here are typical for these species. Wortman et al. [4], who used cover crop species from the Fabaceae and Brassicaceae families, observed apparent antagonism between the mustard, hairy vetch, and field pea when grown in their mixtures; however, we observed no evidence of antagonism between the mustard and legumes. These differences could be due to site-specific differences in climate, soil type, or other factors that varied between the two studies.

The over-yielding we observed with the mixture did not appear to enhance any of the other agroecosystem services typically associated with cover crops. For example, the mixture did not suppress ambient weed abundance compared to the most suppressive component crop (i.e., buckwheat; Figure 3). Similarly, biomass of the surrogate weeds was not lower in the mixture compared to the most suppressive monoculture (Figure 4). These results are in agreement with those of Teasdale and Abdul-Baki [27], who found that cover crop mixtures containing two legumes (hairy vetch and crimson clover, *Trifolium incarnatum* L.) and cereal rye reduced weed biomass compared to the legume monocultures but not the cereal rye monoculture. The trend for lower ambient and surrogate weed biomass in the buckwheat monoculture, suggests that this species is particularly effective in suppression of late spring and summer-emerging weeds; a result that has been observed in previous studies [28].

Despite strong theoretical and empirical support for a link between plant species diversity and agroecosystem stability [16], [29], the over-yielding we observed with the mixture did not enhance stability, either in terms of cover crop biomass or weed suppression, relative to the most stable component monoculture (Figure 5). These results are also in agreement with those reported by Wortman et al. [4] who found that the relative stability of cover crop biomass production was not associated with the number of different legume and brassica cover crop species grown in mixtures. Considered along with that study, our results suggest that greater cover crop functional group richness (i.e., mixtures with four plant families) does not necessarily improve stability. This conclusion is in accordance with recent work by Cardinale et al. [30] suggesting that effects of species diversity on biomass production can be independent of diversity effects on stability.

Our results suggest that of the five cover crop species examined, buckwheat grown in monoculture should be preferred over this specific mixture if a producer’s goal is to produce consistent (spatially and temporally stable) summer cover crop biomass and to maximize summer weed suppression. Cereal rye, which did not produce excessive biomass, did provide a fairly consistent (stable) level of weed suppression across replicates and years, and would likely be preferable to the mixture examined here, if the primary goal was weed suppression.

One of the primary motivations for growing cover crops is to improve growing conditions for a subsequent cash crop [2]. Relative to a monoculture, a cover crop mixture should be expected to contribute residues that vary in quality and biochemical composition, which in turn could affect soil processes (and their microbial drivers) that influence crop growth [31]. We used common oats as a “phytometer” to assess whether the cover crop mixture resulted in carryover effects that would improve cash crop productivity compared to the component species grown as monocultures. We found no evidence to suggest the mixture enhanced oat growth more than the component species, although due to land and labor constraints the carryover study was not conducted following the 2011 study. Therefore, these results should be interpreted with some degree of caution. Despite this caveat, our results are congruent with a recent study showing no difference in crop yields associated with cover crop mixtures differing in the number of legume and *Brassica* species [8], [17]; however, that study did not include cover crop monocultures.

How do we explain the fact that over-yielding with the cover crop mixture resulted in no apparent enhancement of other agroecosystem services relative to the component monocultures? One possible explanation is that the increased yields appeared to be driven primarily by a single species, buckwheat. Therefore, the potential for concomitant effects on other functions was relatively limited. Another possible explanation is the metric used to assess yield response. The LER has primarily been used to measure the yields of cash crops grown in polyculture [21]. When applied to cash crops, the proportional yield is a relevant metric, providing information about the amount of land area that would be required to produce an equivalent yield of each crop in monoculture as can be obtained by growing those crops in mixture, and has important implications for improving the efficiency of agricultural land use [32]. However, when applied to cover crops, a proportional metric such as LER does not align with the purpose of cover crops, as the goal is often to maximize total cover crop biomass per unit area rather than minimize the total land area required to grow a certain number of different cover crop species. Thus, if a particular agroecosystem function, such as weed suppression, is strongly linked to cover crop biomass [10], [11], a cover crop mixture can over-yield but still not “out-perform” the most productive monoculture. Finally, we utilized a limited number of potential cover crop species (five) and quantified only a relatively small subset of the possible agroecosystem services associated with cover crop use in a single season. For example, we did not assess beneficial insect populations, soil-borne disease, or soil organic matter quality and diversity, all of which could be affected by our cover crop treatments and which may manifest over longer time periods of cover crop use [31], [33], [34]. Additional research will be necessary to determine the full range of mixture combinations and cover crop planting niches, and their effects on a wider range of possible agroecosystem functions, including food-web dynamics, biological control, and weed-crop competitive interactions.
